# Intracellular RNA-tracking methods

**DOI:** 10.1098/rsob.180104

**Published:** 2018-10-03

**Authors:** Logan George, Fred E. Indig, Kotb Abdelmohsen, Myriam Gorospe

**Affiliations:** 1 Laboratory of Genetics and Genomics, National Institute on Aging Intramural Research Program, National Institutes of Health, Baltimore, MD 21224, USA; 2 Confocal Core Facility, National Institute on Aging Intramural Research Program, National Institutes of Health, Baltimore, MD 21224, USA

**Keywords:** RNA labelling, mRNAs, noncoding RNAs, post-transcriptional gene regulation, ribonucleoprotein complexes

## Abstract

RNA tracking allows researchers to visualize RNA molecules in cells and tissues, providing important spatio-temporal information regarding RNA dynamics and function. Methods such as fluorescent *in situ* hybridization (FISH) and molecular beacons rely on complementary oligonucleotides to label and view endogenous transcripts. Other methods create artificial chimeric transcripts coupled with bacteriophage-derived coat proteins (e.g. MS2, λN) to tag molecules in live cells. In other approaches, endogenous RNAs are recognized by complementary RNAs complexed with noncatalytic Cas proteins. Each technique has its own set of strengths and limitations that must be considered when planning an experiment. Here, we discuss the mechanisms, advantages, and weaknesses of *in situ* hybridization, molecular beacons, MS2 tagging and Cas-derived systems, as well as how RNA tracking can be employed to study various aspects of molecular biology.

## 1. Introduction

RNA molecules have a broad range of roles in the cell. Coding RNAs (messenger (m)RNAs) serve as templates for the translation of proteins, while noncoding RNAs (including microRNAs (miRNAs) and long non coding RNAs (lncRNAs)) regulate gene expression programmes on many levels [1,2]. RNAs govern all aspects of cell metabolism and thus have been shown to be essential regulators of physiologic and disease processes [3,4].

Recent advances in RNA biotechnology have allowed researchers to interrogate the transcriptome *en masse*. Microarrays are fast and cost-effective methods for quantifying RNA levels in distinct populations, and RNA sequencing (RNA-seq) can further elucidate the identity of the RNAs at the cell or tissue levels [5–7]. Immunoprecipitation (IP) of native or cross-linked ribonucleoprotein (RNP) complexes (RIP and CLIP, respectively) can further identify the targets of a given RNA-binding protein and provide insight into RNA–protein interactions [8]. These methods have transformed the RNA field. However, none of these techniques provide information on the spatial or temporal dynamics of the RNA in the cell, including its location as the RNA is processed, transported, stored, translated or degraded. To study these parameters, researchers must somehow label an RNA of interest and analyse it using microscopy.

Advances in RNA localization at a subcellular level have improved our understanding of viral and neurodegenerative diseases through characterization of RNA-mediated pathological mechanisms. For example, analysis of the trafficking of hepatitis C virus (HCV) RNA revealed that host cells increase the production of type-I interferons by packaging viral RNAs into exosomes; these are then transported to nearby dendritic cells, activating the TLR-7-mediated antiviral response [9]. Other studies revealed that RNA export from the nucleus was inhibited by the formation of toxic protein aggregates [10], an observation that may be relevant to Alzheimer's and Huntington's diseases, for example, as they are driven by the accumulation of toxic proteins [11].

RNA localization is also important for establishing cell polarity and asymmetry during development. Developmental patterning is achieved by restricting mRNA translation to one side of the cell, effectively localizing the respective protein products to subcellular compartments. This is evident in *Drosophila* oocytes, wherein regulation of *bicoid* mRNA localization establishes head and thorax regions in the egg [12]. Similar mechanisms have also been identified in *Xenopus* and zebrafish development [13,14].

In adult mammals, one notable example of long-range mRNA transport is found in neurons in the form of messenger ribonucleoprotein (mRNP) granules [15]. These mRNP granules are stored in ‘translational hotspots’ within the neurons [16]. Neural depolarization triggers the mobilization of storage granules to form actively translating polysomes [17]. The complex biological roles of mRNP complexes have been studied for the past 20 years, revealing important links to neuronal survival and function [15,18,19].

To study the localization and transport of cellular RNA, several methods of RNA tracking have been developed that have revolutionized the field. This review describes the most popular among these techniques; specifically, we discuss methodologies to tag endogenous RNAs by using fluorescent oligomer tags (fluorescent *in situ* hybridization (FISH)) or beacons, to track individual RNAs by making chimeric RNAs bearing tractable elements (e.g. MS2, boxB, RNA aptamers), and to identify endogenous RNAs through a complementary RNA complexed with noncatalytic Cas9/Cas13 (table 1). We also discuss how these technologies are helping to elucidate important RNA biology.

**Table 1.** Overview of RNA tracking techniques. RNA FISH employs antisense oligomers conjugated to fluorophores to track target transcripts in fixed cells. Molecular beacons are conceptually similar, but the addition of a quencher reduces background noise from unbound probes, improving their usefulness in live-cell experiments. MS2 tagging and similar aptamer-based methods involve cloning unique sequences (MS2 hairpins, RNA aptamer sequences, etc.) into the 3′UTR of the transcript of interest; this sequence is then recognized and bound by a fluorescently tagged protein or other molecule using live or fixed cells. RNA tracking with Cas proteins uses catalytically inactive Cas proteins tagged with a fluorescent protein. These fluorescent proteins identify target RNAs based on sgRNA targeting; depending on the Cas protein used, additional oligomers called PAMmers may also be needed.

RNA visualization techniques	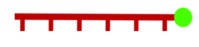 RNA FISH	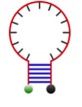 molecular beacons	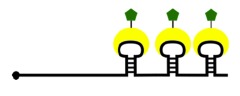 MS2 tagging	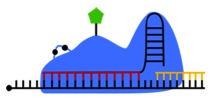 Cas-derived methods
mechanism	antisense oligonucleotides hybridize with target RNAs conjugated fluorescent dyes allow visualization *in situ*	hairpin oligonucleotides bind complementary target RNAs quencher reduces off-target noise	plasmid encoding RNA of interest tagged with MS2 hairpins is transfected into cells MS2 coat proteins with fluorescent tag bind strongly to these hairpins for visualization	deactivated Cas proteins tagged with fluorescent probes target RNA of interest bound to sgRNA sequence
advantages	complementary oligomers create strong target specificity amenable to multiplexing tiling oligomers enhance detection of single molecules	live-cell applications capable of multiplexing low background noise from unbound probes	live-cell applications coat protein only binds MS2-tagged RNAs, minimal off-target signals compatible with protein immunofluorescence	live-cell applications sgRNA creates high specificity delivery by single RNP transfection compatible with protein immunofluorescence
limitations	samples must be fixed during assay fails if binding sequence masked by structure or RBPs difficult to combine with protein immunofluorescence	introduced using toxic membrane permeabilization methods fails if binding sequence on target is hidden by structure or proteins	cannot multiplex requires new plasmid for each RNA tested and optimization of MS2-RNA to MS2-protein ratios introduced using toxic membrane permeabilization methods	cannot multiplex some require PAMmers limited resources available at this time for RNA-targeting sgRNAs and PAMmers.

## 2. RNA fluorescent *in situ* hybridization (RNA FISH) and RNA beacons

*In situ* hybridization is a powerful molecular biology technique that has been ubiquitous in the field of nucleic acids research since its debut in the 1960s [20]. Antisense oligonucleotides (ASOs) with autoradiographic labels such as ^3^H or ^32^P allowed researchers to target DNA or RNA sequences that were complementary to the oligomer, allowing the visualization of these sequences inside fixed cells or tissues [21]. Over the years, this technique evolved with the introduction of new methods of detection, such as gold labelling in conjunction with electron microscopy or enzyme-linked chromogenic reporters [21].

### 2.1. RNA FISH

**Figure 1.**
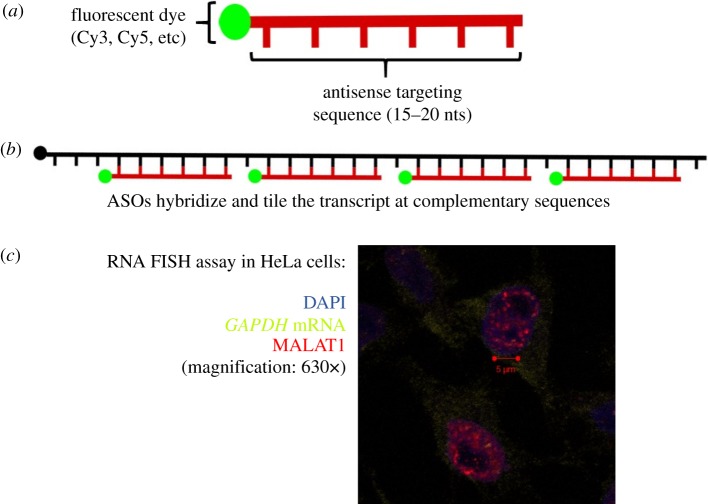
RNA FISH. (*a*) Short oligomers are synthesized with a targeting sequence that is antisense to the RNA of interest. Antisense oligomers (ASOs) are conjugated with fluorescent dyes that can be excited with confocal or fluorescent microscopy. Presently, RNA FISH oligomers are provided as a ‘cocktail’ of different ASOs that can tile the target RNA. (*b*) ASOs hybridize to the target RNA with the antisense targeting sequence. By tiling the RNA of interest with ASOs, the signal is amplified to facilitate detection of low-copy or diffuse transcripts in the cell. (*c*) Example of RNA FISH in HeLa cells imaged with confocal microscopy (unpublished image from the authors). *GAPDH* mRNA (light green), tagged with Quasar 670 ASOs, is detected in the cytoplasm. *MALAT1* (red), tagged with Quasar 570 ASOs, is localized in nuclear speckles.

FISH was introduced in 1980 by the van Duijin lab [22], and was widely adopted to study nucleic acid localization during development, viral infection and other cellular and molecular responses. Initially developed to identify genomic DNA regions that were specifically complementary to synthetic antisense-RNA probes (figure 1*a*), FISH was soon adapted to detect various types of RNA molecules [23–25]. A key advance in RNA FISH technology was the development of single-molecule FISH (smFISH), where multiple consecutive fluorescent probes hybridize the RNA in a ‘tiling’ fashion, and the presence of multiple probes amplifies the signal (figure 1*b*) [26]. This technique is particularly useful for detecting low-copy transcripts, as the high number of probes bound to a transcript can light up a single RNA molecule with multiple fluorophores.

While RNA FISH allows tracking of RNA molecules in cells, it is also a valuable tool for quantifying the spatial distribution and copy number of a target RNA. RNA-seq and reverse transcription (RT) followed by real-time quantitative polymerase chain reaction (qPCR) assays can directly quantify transcripts in tissues or single cells [6,27,28], but they typically do not inform about the localization and expression of a specific RNA in subcellular compartments such as the nucleus. Thus, quantification by RNA FISH is uniquely useful for determining copy number and location. Today, RNA FISH is considered one of the gold standards for RNA localization.

FISH has been used since its advent to study cellular RNA trafficking. FISH analysis of individual *β-actin* (*ACTB*) mRNAs revealed that their localization in the leading lamellae in fibroblasts contributes to cell motility [29]. Similarly, *β-actin* mRNA transport via zipcode-binding protein 1 in neural growth cones regulates the direction of axonal growth, as demonstrated by FISH coupled with immunostaining [30]. The experiments mentioned above involving trafficking of the HCV RNA [9] and nuclear RNA export inhibition [10] were also conducted using FISH.

RNA FISH can be multiplexed to identify different transcripts in the same sample. Kosman and colleagues [31] used this approach to detect localization of five unique transcripts in *Drosophila* embryos. The main limitation of this approach is the number of available microscope filters, as the emission spectrum of each fluorophore is unique. Although FISH methods are ideal for *in situ* analysis of fixed samples, they are not amenable to visualization of RNA in live cells. The need to fix the samples for analysis makes it impossible to study subcellular RNA dynamics in real time. In addition, the fixation and preparation of samples with strong chemicals like paraformaldehyde or hydrochloric acid can lead to biochemical alterations within the cells, disrupting cellular structures or denaturing proteins and organelles [32]. To alleviate these concerns and enable the analysis of live cells, molecular beacons, which employ methods like FISH, have been developed to visualize RNA in fixed and live cells.

### 2.2. RNA molecular beacons

**Figure 2.**
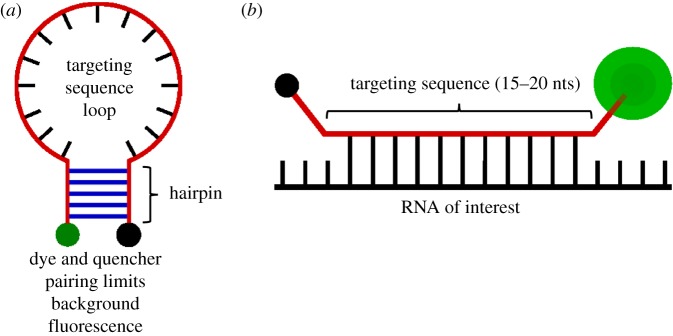
Molecular beacons. (*a*) The most common molecular beacon structure is a stem-loop style probe. The stem-loop structure brings the fluorescent dye close to the quencher molecule. This quencher absorbs the energy emitted by the dye and releases it as heat, reducing fluorescent background noise. The loop contains the targeting sequence that will hybridize with the RNA of interest. (*b*) The hairpin dissociates when the targeting sequence hybridizes to the target RNA. This separation moves the fluorescent dye out of range of the quencher, allowing emission of detectable fluorescence.

The basic principle of molecular beacons is the same as FISH: to employ fluorescently tagged oligos that bind complementary transcripts of interest in the cell. The main difference lies in the coupling of the fluorophore to a quencher [33]. Quenchers are compounds that absorb the energy emitted by the fluorophore and dissipate it as heat instead of light, dramatically reducing the background signal of unbound probes [33]. The most frequently used form of molecular beacons is the stem-loop (figure 2*a*) [34]. The target sequence (15–20 nts) forms a loop flanked by a palindromic sequence on each end; the 5′ end is often conjugated to a fluorophore, while the 3′ end has a quencher attached [33]. When the beacon is not bound to a complementary target, the palindromic sequences form a hairpin stem, bringing the fluorophore and quencher close enough for the quencher to suppress the fluorescence; however, binding to the target sequence causes the hairpin and the attached fluorophore and quencher to separate, resulting in the emission of fluorescence (figure 2*b*) [33]. The absence of signal when beacons are unbound reduces noise. Thus, beacons can be introduced into live cells, allowing one to observe their respective RNA targets in real time. Since RNA targets are bound by antisense fluorophore-tagged oligomers, multiplexing with molecular beacons is possible [35]. Beacons may be introduced into cells using electroporation, microinjection, toxin-mediated membrane permeabilization, or even through heavy metal bioballistic gas guns. Each method has its specific limitations, but collectively they enable RNA visualization in various cell models [36,37].

### 2.3. RNAscope

While RNA visualization is commonly used in basic biomedical research, these techniques are not widely used in diagnostics, where RT-qPCR analysis is preferred for the detection of oncogenic or viral transcripts [38]. However, as described above, PCR-based approaches lack spatial resolution, and their sensitivity can be disrupted by heterogenous tissue samples. RNAscope was developed to improve the diagnostic capabilities of RNA detection. The technique represents an improvement on single-molecular FISH that facilitates detection of low-copy transcripts in tissue samples [39]. Although it was designed for RNA detection in paraffin-embedded tissue samples, it can be adapted to basic cell and molecular research as well.

**Figure 3.**
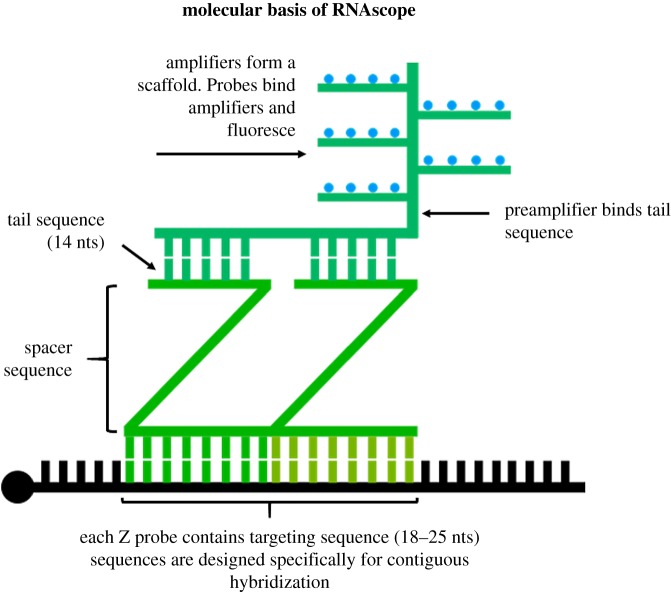
RNAscope probe design. Two Z-shaped probes bind adjacent sequences on the RNA of interest, forming a platform-like structure across the tail sequences. Here, the preamplifier binds, and can only bind when two Z probes are bound. Amplifiers bind the preamplifier, serving as scaffolds for the fluorescent probes to bind. Each set of Z probes provides binding sites for hundreds of fluorescent probes, dramatically amplifying fluorescence on a single molecule. Z probes can be tiled along an RNA molecule to further improve detection.

Instead of using antisense oligomers directly conjugated to a fluorophore, RNAscope amplifies fluorescence by first assembling a scaffold on the RNA of interest, the foundation of which consists of Z-shaped probes (figure 3). These probes have a targeting sequence of 18–25 nucleotides that are complementary to the target. Attached to this probe is a spacer sequence, connected to a 14-nucleotide long tail sequence. Targeting sequences are designed so that two probes will hybridize directly adjacent to each other; similarly, the two tails form a continuous 28-nucleotide hybridization site. The coupled tails form a sequence recognized and bound by the preamplifier, a backbone-like structure containing 20 binding sites. These binding sites are complementary to amplifiers, which attach and complete the scaffold. Once the amplifiers are bound, up to 20 dye molecules can bind each amplifier to produce a robust fluorescent signal within the cell or tissue [39]. Given that many common dyes, such as Alexa 488 or Alexa 647, are compatible with RNAscope, imaging can be done on most fluorescent or confocal microscopes.

Z probes must bind directly adjacent to the target transcript to fully assemble the RNAscope scaffold, reducing much of the background fluorescence and improving the signal-to-noise ratio substantially. In addition, one set of Z probes can contain up to 400 dyes, and therefore designing multiple Z probes to tile the RNA of interest dramatically increases the ability to detect single RNA molecules. When detecting rare or isolated transcripts in large tissue sections, one set of RNAscope probes will provide a strong signal for detection; in contrast, standard RNA FISH would require tiling of oligomers, which entails designing dozens of oligomer sequences for a single target RNA. Depending on the length of the RNA of interest, this may not even be possible, making RNAscope a more suitable approach. The short length of the Z-probe targeting sequences (two 18–25 bp sequences) makes them suitable for targeting miRNAs, and perhaps circular RNAs at splice junctions as well. RNAscope can be multiplexed to up to four independent targets in a single sample [39], limited mainly by the number of available dyes. However, this technique is mainly designed for use in tissue, and as such, it is currently restricted for use in paraffinized samples.

## 3. Bacteriophage-derived RNA tags

The methods described so far rely on complementary base-pairing of oligomers to the target transcript. One drawback of these approaches is that if the target sequence is unavailable, either because it is embedded in a hairpin, bound to another nucleic acid, or associated with an RNA-binding protein (RBP), then hybridization cannot occur [40]. In addition, the abundance of endogenous transcripts might be low, further limiting the sensitivity of the approach. To overcome these limitations, researchers have developed labelling systems based on the addition of a bacteriophage-derived RNA tag to study the dynamics of an individual RNA in live cells. First demonstrated in yeast [41], the technique has been developed to study RNA mobilization within cells and interaction with different *trans*-acting factors, including RBPs, miRNAs and lncRNAs [42,43].

### 3.1. MS2/MS2-BP and boxB/λN

**Figure 4.**
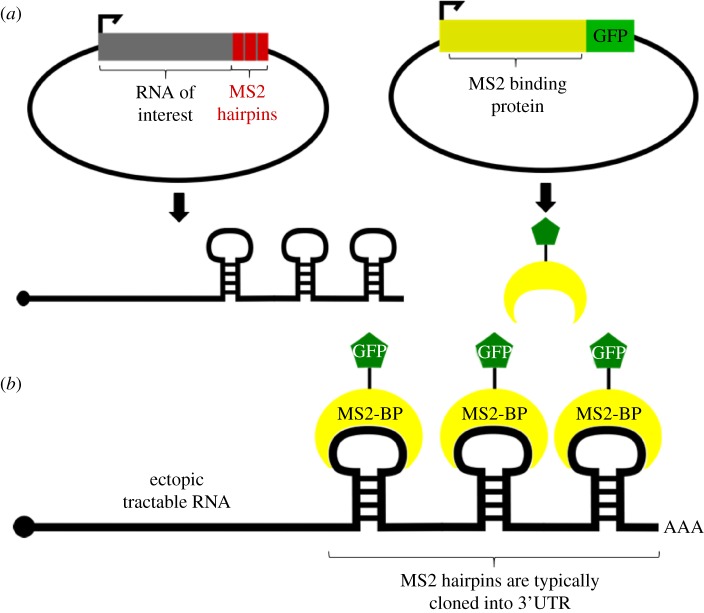
MS2 RNA tagging. (*a*) MS2 experiments require generation of two constructs. One construct encodes the RNA of interest (or a certain region, such as only the 3′UTR) with MS2 hairpins encoded downstream. When transcribed, the MS2 hairpins will form in the 3′ end of the transcript and be recognized by the MS2 binding proteins (BPs). MS2-BPs are expressed from the other construct and generally include a fluorescent protein such as GFP. (*b*) Following transfection, both constructs are transcribed and the MS2-BPs are translated. The ectopic transcript of interest is detected and bound by the fluorescent MS2-BPs. The number of hairpins encoded in the plasmid will determine the number of MS2-BP binding sites; cloning more hairpins will amplify the signal and improve detection. Since unbound MS2-BPs will still fluoresce in the cell, determining proper plasmid transfection ratios is essential for maximizing signal-to-noise ratios.

The MS2 tagging system is based on the coat protein of the MS2 bacteriophage, which contains an RNA-binding site with high binding affinity for RNA stem-loop structures found only in the bacteriophage RNA [44]. Bacteriophages normally use this coat protein to ensure encapsidation of the viral RNA genome [45]. As these hairpin structures do not exist in mammalian RNA, the MS2 coat protein does not interact with proteins or RNAs synthesized by the cell. However, introducing a plasmid that transcribes a transcript of interest bearing multiple MS2 hairpins (often inserted in the 3′-untranslated region (UTR) of the chimeric mRNA) enables the coat protein to bind these exogenous transcripts with high affinity and specificity (figure 4). Fusing a fluorescent protein such as GFP to the MS2 coat protein (generically MS2-XFP) further enables the easy observation of the RNA in the cell [39]. The signal may be amplified by including additional hairpins to the 3′UTR, thereby increasing the number of MS2-binding sites. Most constructs have 6 to 24 MS2 hairpins attached, to form a longer chimeric RNA. A similar system has been developed to exploit another bacteriophage tractable tag, the boxB sequence, which is recognized by the bacteriophage protein λN. Tagging methods have been developed that track a λN-XFP fusion protein interacting with a boxB motif inserted in a chimeric transcript of interest [46], but they have been used less frequently than the MS2 system in recent years.

The MS2 system has been employed to study RNA localization in a multitude of biological contexts. Sheth and Parker [47] used it to demonstrate that yeast RNA decay intermediates are localized in cytoplasmic processing bodies (PBs). Similar experiments were used later to gain evidence that miRNAs and Argonaute suppress mRNA translation in mammalian PBs [48]. Live *Drosophila* oocytes expressing ectopic *nos-MS2* and MS2-GFP were used to demonstrate the timing of mRNA transport in developing oocytes and the role of the cytoskeleton in transcript trafficking [49]. MS2-tagged *Pkp4* 3′UTR constructs were used to demonstrate the preferential recruitment of RNAs found in adenomatous polyposis coli-containing ribonucleoprotein (APC-RNP) complexes to granules that contained mutant forms of the protein FUS and have been linked to amyotrophic lateral sclerosis [50,51]. Recent research by Morisaki and colleagues [52] used MS2-mRNA tagging as part of an experiment to track single-transcript translation *in vivo*. mRNAs were tracked with MS2, and the nascent FLAG-tagged polypeptides were tagged with fluorescently labelled anti-FLAG Fab fragments. Importantly, neither the tags nor the fluorescent proteins disrupted normal protein or transcript distribution.

In each of these studies, RNA localization relative to subcellular structures was tracked by its association with fluorescently tagged proteins and/or immunostaining of protein markers. The MS2 system enables studies of association of an RNA of interest with an endogenous protein more easily than FISH, since fixation is often not required.

### 3.2. Advantages and drawbacks of bacteriophage tags

MS2 tagging is best suited to track RNA in live cells, a virtually impossible task with FISH. An important advantage of the MS2 system is that, in theory, the MS2 RNA tag should not impact upon the natural function of the endogenous RNA. By contrast, when a transcript of interest is detected via associated (tagged) antisense oligomers, there is a chance that the oligomers may cover an RBP binding site, disrupting normal processes such as trafficking or loading into an RNP complex. Conversely, in live cells, antisense oligomers may directly interfere with mRNA translation or other RNA functions. In addition, in fixed cells, antisense oligomers recognizing the same site as an RBP may be masked when the RBP is bound, thereby preventing visualization. While careful oligomer design can avoid some of this interference, unknown interactions between oligomers and RBPs can create further artefacts. By contrast, since MS2 hairpins are generally added to the distal end of the 3′UTR, and the coat protein will bind these hairpin repeats exclusively, there is less concern of accidentally interrupting cellular processes during experimentation.

Despite these advantages, there are limitations to this technique as well. While cloning extra hairpin repeats may enhance detection, replication and transcription of long sequences of palindromic DNA repeats can be unstable and lead to slips in the DNA polymerase, which may result in loss or extra insertions of these hairpins [53]. In addition, one must create a new construct for each chimeric transcript of interest, which requires separate cloning efforts for each construct. Unbound MS2-XFP molecules can also cause high levels of background noise. To improve signal-to-noise ratios, cells must receive a ‘correct’ amount of MS2-tagged RNA relative to MS2-XFP plasmids [54]. The MS2 system requires transfection or electroporation of a minimum of two plasmids (one expressing the MS coat protein, the other the MS2-tagged RNA), and the optimization of transfection can be challenging. Finally, given that MS2-XFP will bind any RNA with the MS2 hairpin structure, two different MS2-RNAs expressed in a single cell cannot be distinguished, so the system is not amenable to multiplexing. If detection of multiple RNAs is required, then MS2 detection methods must be combined with other systems like molecular beacons (above), the boxB/λN system, or perhaps a CRISPR/Cas-derived method (below).

## 4. Cas-derived systems and live-cell RNA tracking

CRISPR/Cas-based technologies are well known for their immense potential in genome editing and genetic engineering [55]. The technology was recently expanded to include fluorescently tagged Cas proteins to bind and track RNA in living cells.

### 4.1. Cas9

**Figure 5.**
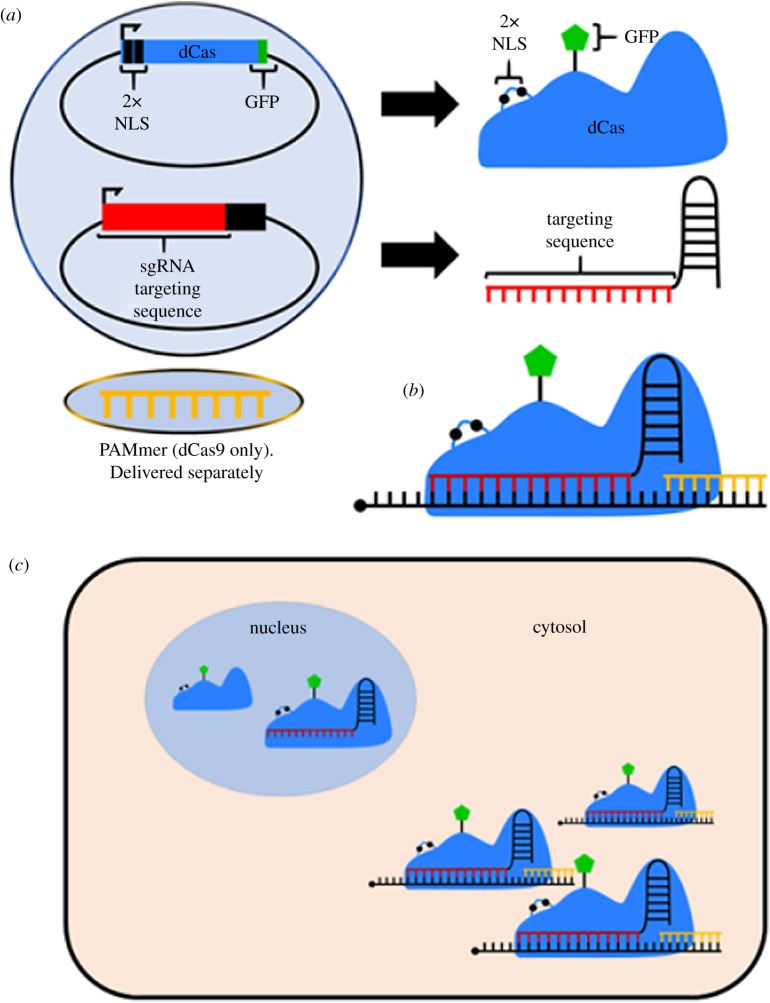
dCas RNA tracking methods. (*a*) Two plasmids are required for dCas experiments. One plasmid encodes the catalytically inactive Cas protein, with mutations to inhibit any nuclease activity. Cas is also modified with two nuclear localization signals and a fluorescence domain, such as GFP or mCherry. The other plasmid contains the sgRNA scaffold, and will synthesize the sgRNA when transcribed. The targeting sequence in determined by the sequence of the sgRNA. If dCas9 from *S. pyogenes* is used, a separate oligomer called a PAMmer must also be transfected into the cell. The PAMmer is not necessary when using dLwaCas13a protein. (*b*) Structure of a fully assembled dCas9 complex. The target RNA is identified by the sgRNA targeting sequence. dCas9 requires a PAM sequence to be present on the off-target strand. Since RNA is single stranded, the PAMmer binds downstream to provide a PAM sequence for dCas9 to recognize and bind. (*c*) The NLSs improve signal-to-noise ratio by sequestering unbound dCas proteins. When dCas is free, or only bound to the sgRNA, the protein is held in the nucleus due to the double-NLS tag. Only fully assembled complexes, which consist of dCas, the sgRNA, the target RNA and the PAMmer (if necessary), are exported to the cytoplasm. In principle, any fluorescence observed in the cytoplasm is true signal.

The most widely studied Cas protein is Cas9 from *Streptococcus pyogenes* [56,57]. Recently, the Yeo lab demonstrated that catalytically inactive Cas9 (dCas9) may be exploited to track endogenous mRNAs in the cytoplasm during the assembly of stress granules, cytoplasmic ribonucleoprotein aggregates that form transiently in response to damaging signals (figure 5) [58,59]. Being catalytically inactive, dCas9 is unable to cleave the target RNA, and instead remains bound to it; however, it only binds nucleic acids that present a protospacer adjacent motif (PAM). For DNA, the PAM must be on the non-target strand (reviewed in [60]); since mammalian RNA is single-stranded, Yeo and colleagues [58] developed PAMmers, short oligonucleotides that contain a sequence complementary to the target RNA, effectively replacing the non-target strand. In this manner, PAMmers associate with the dCas9–sgRNA complex and bind to the transcript of interest. Using FISH as a control, dCas9 was shown to bind mRNA with high specificity and without affecting its transcription, half-life or translation [58]. When not bound to sgRNA, the fluorescent dCas9 proteins are restricted to the nucleus, due to dual nuclear-localization signals (NLSs) at the C-terminus, reducing cytoplasmic signal when not actively in use. The dCas9 system demonstrated less noise than FISH and was effective in tracking endogenous RNA targets in live cells [58].

Limitations of this method include the necessity of developing PAMmers, which increases the complexity of implementation of this method, requiring the transfection of (i) a plasmid expressing dCas9 (which is quite large, 10–14 kbp), (ii) the sgRNA, and (iii) the PAMmer oligomers (figure 5*a*). Exposing cells to this volume of foreign nucleic acids could be stressful [61]. More complex experiments, such as those requiring additional fusion proteins to study colocalization, face further challenges of optimization of DNA quantities and ratios to maintain acceptable transfection efficiencies while preserving cell homeostasis. Given that this RNA tracking system was reported recently, there are no resources available to design sgRNAs or PAMmers targeting a specific RNA, so its implementation has been slow. It may be possible to overcome some of these limitations by delivering the RNA-tracking components directly to the cell as pre-formed dCas9–sgRNA RNPs. The pre-assembly of PAMmers, sgRNA and Cas proteins to streamline the targeting has already been demonstrated for genome editing, so it may translate easily to dCas–sgRNA complexes as well [62].

### 4.2. Other Cas proteins

Another Cas system, Cas13a, isolated from *Leptotrichia wadei* (LwaCas13a) [63], has recently been proposed for tracking RNA in live cells. LwaCas13a is capable of targeting ssRNA, thus eliminating the need for PAMmers and making sgRNA the only determinant for RNA targeting. Fluorescently tagged, catalytically inactive LwaCas13a (dLwaCas13a-XFP) effectively tracked endogenous RNAs in living cells. As with the dCas9 system, the dLwaCas13a system has an NLS, which restricts the fluorescent dCas13a protein to the nucleus unless bound to both the sgRNA and the target RNA, upon which it is exported to the cytoplasm [63]. This technique appears to be superior to the dCas9 system, as dLwaCas13a does not require a PAMmer and thus the smaller ‘transfection load’ allows greater efficiency and more complex experimental designs. Online resources are being developed for data analysis [63]. However, neither method can currently multiplex endogenous tracking targets, since each dCas9 or dCas13a complex will bind sgRNAs indiscriminately

The use of dLwaCas13a technique has several shortcomings. The first is that several sgRNAs need to be tested to optimize sequence recognition. With the catalytically active LwaCas13a, the guide position had a dramatic impact on the level of knockdown, and shifting the guide position a few bases reduced gene knockdown [63]. It is possible that this limitation may also be seen with the catalytically inactive variant, reducing its specificity. In addition, binding of the sgRNA–Cas complex to the target transcript may disrupt RBP binding, should sgRNA target sequence overlap with the RBP binding site.

A recent study [64] demonstrated that Cas9 from *S. aureus* (SauCas9) can bind and cleave ssRNA, requiring only the sgRNA, not a PAMmer. While the study only demonstrates the cleavage capacity of SauCas9, an inactive variant with a fluorescent tag would likely be capable of binding and tracking RNA in live cells. In this case, the tagged variant would function in a similar manner to the dLwaCas13a system. Given how well characterized Cas9 systems are, this may potentially become a next robust method of Cas-derived RNA tracking.

## 5. Additional considerations

We have described several popular RNA visualization methods that rely either on fluorescent RNAs (FISH, molecular beacons, RNAscope) or fluorescent proteins recognizing unique RNA tags (MS2/λN and catalytically inactive Cas).

### 5.1. Further strengths and limitations

As discussed above, FISH and molecular beacons employ fluorophore-labelled antisense oligos that bind target RNAs based on complementary sequences [22,26]. The use of FISH is limited to fixed cells, while molecular beacons overcome this limitation through coupled quenchers that reduce background noise when deployed to living cells. Neither method, however, can be used in complex living organisms, as there are no effective means of introducing the probes into large regions of living tissue, such as the brain; however, molecular beacons may be used in live single-cell organisms. If used in fixed samples, both hybridization techniques are applicable to cells and tissue samples, but RNAscope is predominantly used for detecting transcripts in paraffin-embedded tissues for clinical diagnostics [39]. Since both techniques bind complementary sequences on target transcripts, their success depends on the accessibility of these sequences; if the target is hidden in structural motifs such as hairpins, or covered by an RBP, then both techniques lose sensitivity. The use of tiling oligomers can increase detection, as more fluorophores can interact with the transcript of interest. Given that the RNAscope method inherently amplifies the signal from a single probe without the need of tiling, they are better suited for detection of rare or isolated transcripts. FISH, RNAscope and molecular beacons are also the only techniques discussed here that can detect multiple RNAs (known as multiplex detection) in a single assay if probes are labelled with different fluorophores.

RNA tagging using chimeric fluorescent RBPs relies on the addition of RNA sequences in *cis* (e.g. MS2, boxB) or *trans* (gRNA). When using bacteriophage RNAs (MS2, boxB), one plasmid expressing the fluorescently tagged MS2 or λN proteins and another plasmid expressing the RNA of interest tagged with MS2 or boxB hairpin repeats are introduced into cells. The fluorescent proteins then bind the RNA tags, permitting the localization of exogenous transcripts in live cells. Since this assay does not depend on complementary oligomers, there is less risk of RNA structure or binding proteins decreasing the sensitivity and lower concern with background. The sensitivity for a single transcript can be increased by cloning additional MS2/boxB tags into the chimeric RNA, creating more RBP recognition sites. Bacteriophage-derived RNA tracking methods require transfection of two plasmids (the phage-tagged RNA and the tag-binding protein), and transfection ratios must be optimized to achieve an acceptable signal : noise ratio. The plasmids are typically introduced through transient transfection without stable incorporation into the genome, so this method is mainly useful for cultured cells and single-cell organisms, such as yeast. Following transfection and assay execution, samples may be fixed for static imaging as well.

Similarly, fluorescent, nuclease-deficient Cas proteins (e.g. dCas9-GFP) can be directed by sgRNAs to target RNAs in live cells in the presence of an additional oligomer, a PAMmer [58,63]. A variation of this method includes use of fluorescent dLwaCas13a, which also requires a sgRNA, but does not need a PAMmer. The NLSs in Cas proteins restrict unused Cas proteins to the nucleus, and only fully formed Cas–target complexes are exported to the cytoplasm, reducing cytoplasmic noise and increasing sensitivity. Whether MS2, λN, or Cas proteins are the chosen tagged fluorescent proteins, the need for plasmid transfections increases the complexity of the assay and may prevent the testing of additional components (e.g. siRNAs or additional plasmids) in these cells. Other limitations include detection of multiple target RNAs at the same time using the same approach, analysis of cells that cannot be transfected, and analysis of RNA in archival samples. Fluorescent Cas-derived systems face many of the same limitations as the bacteriophage-derived methods: the need to transfect multiple plasmids, the limitation of use in living cells or single-cell organisms, and the restriction to track single transcripts. It does not, however, require extensive optimization of transfection, as the dual-NLS tags on the fluorescent Cas proteins will restrict them to the nucleus until the entire complex forms. This feature reduces the background signal in the cytoplasm and facilitates the detection of *bona fide* transcript signals [63,64].

### 5.2. Fluorescent RNA aptamers

RNA aptamers are remarkably specific RNA structures that are capable of binding myriad targets [65]. Aptamers are generated through artificial selection, a process known as SELEX (systematic evolution of ligands by exponential enrichment), allowing identification of RNAs that bind many ligands, such as proteins, other RNAs, and even small molecules like fluorophores [66].

**Figure 6.**
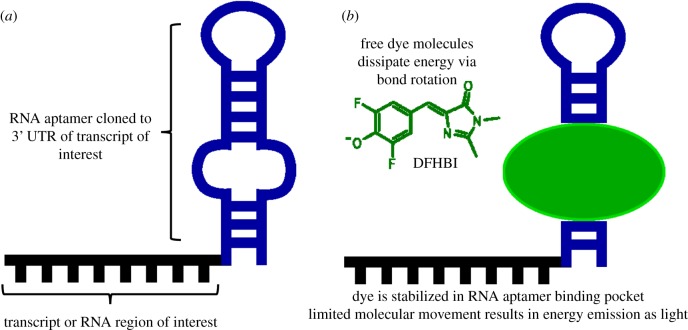
Fluorescent RNA aptamers. (*a*) Structure of an RNA aptamer. Following SELEX for an aptamer that binds the desired dye, this sequence is cloned into the 3′UTR of the transcript of interest. (*b*) Most dye molecules, such as DFHBI (Spinach), do not fluoresce when not bound to the aptamer. Energy is released through bond rotation and other molecular motion. When the dye is bound by the aptamer, motion is restricted. Therefore, energy must be emitted as light, improving signal-to-noise ratio and confidence in molecular detection.

These aptamers are chosen by rounds of artificial selection for RNAs that bind dyes such as the synthetic GFP mimic DFHBI (Spinach) or thiazole orange (TO) derivatives. When these dyes are not bound to aptamers, excited energy is released through molecular movement, such as bond rotation [67]. However, the dyes cannot move when bound to the RNA aptamer, so this energy must be released through light emission (figure 6). This process inherently reduces the background noise produced by free dyes, which overcomes one of the biggest challenges posed by bacteriophage-derived RNA tagging systems. Once the aptamer sequence has been identified through SELEX, this sequence can easily be cloned into a plasmid to produce the aptamer.

Application of these aptamers is remarkably flexible. The aptamer sequence can be cloned into the 3′ region of an RNA of interest, similar to an MS2 hairpin, but the sequence is much shorter, generally in the range 20–80 nts [67]. Once cloned, these tagged RNAs can be used in both fixed- and live-cell experiments. For fixed-cell experiments, transfected cells express the plasmids for several hours to days, then they are fixed and stained with the dye of choice [68]. These samples are still suitable for immunofluorescent staining as well, making the RNA aptamer approach compatible with analysis of RNA–protein complexes. Live-cell experiments involve transfection of RNA-aptamer transcripts that have been produced *in vitro* and pre-incubated with dye [68]. Following aptamer-dye transfection, transcripts can be tracked *in vivo* with minimal background signal.

Despite the introduction of fluorescent Spinach RNA aptamers in 2011 [69], there has been little use of fluorescent RNA aptamers in the RNA field. Cytotoxicity caused by malachite green aptamers and poor signal-to-noise ratios from Spinach aptamers have been limitations to early RNA aptamer technology [67]. Recently, however, improvements on an existing RNA Mango aptamer have made the aptamer approach to RNA visualization more appealing [68]. These RNA Mango aptamers bind TO-biotin, where the TO dye is conjugated to a biotin molecule by a polyethylene glycol chain, with nanomolar affinity. The high affinity, combined with the low background noise of unbound dyes, makes the RNA Mango system effective for localizing RNAs of interest in fixed or live cells. Beyond imaging, RNA aptamers have been used as fluorescent biosensors, able to detect small molecules like ADP or potassium ions [70,71]. Since TO is biotinylated, this system can also be used to purify the RNAs and proteins binding TO [72]. RNA Mango is fairly inexpensive, but investigations into multiple RNAs of interest will require cloning of new constructs to introduce the aptamer sequence to the target RNA 3′UTR. Resources for RNA Mango aptamers are already available, and given its versatility, it is likely that this technology will see widespread adoption soon.

### 5.3. Fluorescent *in situ* RNA sequencing

While RNA FISH can provide insight into transcript quantity, detection is limited by the targeting sequence chosen. Accordingly, antisense probes may not detect splice variants or transcripts with single nucleotide polymorphisms (SNPs). Recent work from the Church lab has introduced novel methods for fluorescent *in situ* sequencing (FISSEQ) of RNAs in fixed cells and tissues [73]. Samples are fixed onto slides and the RNA is converted to cDNA via *in situ* reverse transcription. During circularization, amines incorporated into the cDNA are used to cross-link and prevent diffusion. The cDNA is circularized and amplified by rolling-circle amplification; cross-linking of amines produces cDNA libraries *in situ*. The cDNA is then subjected to sequencing through SOLiD sequencing, a sequence-by-ligation approach (reviewed in [74]).

This technique preserves the cytoskeleton and overall sample structure, and it can detect biologically active RNAs, so researchers can detect functional differences between cell types or regions of tissue. It can be used in cells, formalin-fixed tissues, *Drosophila* embryos, and organoids derived from induced pluripotent stem cells [73]. The entire procedure is performed on a confocal, wide-field epifluorescent or spinning disk microscope; however, sequencing can take several weeks, and this method may not be available to researchers who use core microscope facilities. The authors initially achieved approximately 200 mRNA reads per cell, but suggested optimization that could increase this yield to approximately 5000 reads per cell, primarily through depletion of endogenous rRNAs [73]. At the time it was developed, the technique was able to detect up to 8102 genes, as seen in fibroblasts in a wound-healing assay [75]; no technique described here can determine localization of this many transcripts at once. The authors indicated that FISSEQ may not accurately detect transcripts that are bound to RBPs or locked into complexes, likely because any such complexes become fixed during sample preparation.

### 5.4. Studying RNA localization to understand RNA function

Given their functional diversity, RNA studies have taken centre stage in cellular biology. mRNAs encode proteins needed for cellular processes, while non coding RNAs can perform structural and regulatory functions, such as chromatin organization, ribosome assembly, and transcriptional control, post-transcriptional RNA regulation and post-translational control of protein levels and function [76–78]. The advent of new methods to study the transcriptome, particularly RNA-seq, has uncovered RNAs involved in many physiological processes and pathologies [9,11,12,79,80]. While we can gain extensive information on RNA identity and abundance, we lack the spatio-temporal resolution to understand how RNA molecules interact with cellular machineries and structures. In this regard, the use of RNA tracking techniques such as those described here can begin to address these critical questions.

One area that may benefit from RNA tracking is the field of extracellular vesicles (EVs, including exosomes and microvesicles) [81,82]. These tiny membrane-enclosed structures were once thought to play a role in eliminating cytoplasmic components [83], but have recently been implicated in cancer metastasis, innate immunity and delivery of therapeutic molecules [9,84,85]. They contain many proteins and nucleic acids, including miRNAs (e.g. let-7) and lncRNAs (*MALAT1*) [86,87], but little is known about how their cargo is sorted, as well as how EVs are trafficked in and out of cells [88]. Tracking of RNAs to follow their journey from synthesis to packaging followed by secretion and eventual delivery to recipient cells may provide important insight into the machinery and pathways involved in EV metabolism.

### 5.5. RNA localization in disease processes

RNAs of many types (mRNAs, miRNAs and lncRNAs) are known to play key roles in cancers [79,80], and have been used as prognostic markers. For example, the lncRNAs *MALAT1* and *HOTAIR* are metastasis markers in cancer such as lung, breast and nasopharyngeal carcinomas [89–91]. Although they are potential targets for therapeutic intervention in cancer, their specific role in malignancies is unknown, and hence designing small molecules that will disrupt or restore their function is challenging [92]. It is difficult to intervene upon potential oncogenic targets if their cancer-causing function is not fully known [93,94]. Therefore, understanding the spatio-temporal roles of these RNA molecules is a key step towards distinguishing healthy from cancerous cells. Drug screens may also uncover small molecules that target the RNAs and disrupt their function, and thus it may be beneficial to observe the function of these transcripts before and after treatment to understand the effect of the intervention.

### 5.6. Future directions

Outside of biomedical applications, there are many mysteries surrounding RNA, particularly non coding RNAs. The project ENCODE (Encyclopedia of DNA Elements) aims to identify all functional regions in the human genome and characterize their purpose in the cell [95]. This project revealed that approximately 75% of the human genome is transcribed, while a mere 1.22% of the genome consists of protein-coding exons [95,96]. In addition to challenging the ‘junk DNA’ hypothesis that had existed for years, the sheer volume of noncoding RNAs transcribed at some point in some tissue suggested that many of these transcripts serve some function. Understanding the biological value of these uncharacterized transcripts includes elucidating the subcellular compartments in which they reside. The localization of a noncoding RNA in the nucleus, for instance, may suggest a role in gene organization, transcription and/or early processing. The localization of a noncoding RNA in the cytoplasm may suggest a role in the regulation of stability, translation, storage, and/or mobilization of mRNAs or other cytoplasmic molecules. Further evidence will be needed to fully characterize the impact of noncoding RNAs, but understanding their spatial distribution is an unbiased first step in elucidating their functions.

Despite important advances in the field, however, there are still some restrictions to the application of RNA tracking methods. The systems described here generally require a known RNA sequence, either by creating complementary nucleic acids that seek out the transcript of interest, or by developing vectors that encode chimeric (tractable) versions of the RNA. Therefore, most of the current tracking technologies are not adequate for discovering new RNAs and are limited to studying the function of known RNAs.

There is rising recognition that coding and noncoding RNAs play pivotal functions in all cellular processes, including chromatin organization, transcriptional control, regulation of post-transcriptional events (mRNA transport, stability, storage, and translation), and post-translational processes like protein stability and multiprotein assembly. Along with these expanding functions, evidence is also accumulating that RNA dysregulation can lead to major disease categories including cancer, neurodegeneration, cardiovascular disease and metabolic syndrome. As our ability to track RNAs improves, we will gain insight into their mechanisms of action, expanding our functional understanding of RNAs and enabling the development of therapeutic venues.

## Data accessibility

This article has no additional data.
